# Rapid Interhemispheric Switching during Vocal Production in a Songbird

**DOI:** 10.1371/journal.pbio.0060250

**Published:** 2008-10-14

**Authors:** Claude Z. H Wang, Joshua A Herbst, Georg B Keller, Richard H. R Hahnloser

**Affiliations:** Institute of Neuroinformatics UZH/ETH Zurich, Zurich, Switzerland; University of Minnesota, United States of America

## Abstract

To generate complex bilateral motor patterns such as those underlying birdsong, neural activity must be highly coordinated across the two cerebral hemispheres. However, it remains largely elusive how this coordination is achieved given that interhemispheric communication between song-control areas in the avian cerebrum is restricted to projections received from bilaterally connecting areas in the mid- and hindbrain. By electrically stimulating cerebral premotor areas in zebra finches, we find that behavioral effectiveness of stimulation rapidly switches between hemispheres. In time intervals in which stimulation in one hemisphere tends to distort songs, stimulation in the other hemisphere is mostly ineffective, revealing an idiosyncratic form of motor dominance that bounces back and forth between hemispheres like a virtual ping-pong ball. The intervals of lateralized effectiveness are broadly distributed and are unrelated to simple spectral and temporal song features. Such interhemispheric switching could be an important dynamical aspect of neural coordination that may have evolved from simpler pattern generator circuits.

## Author Summary

As for all vertebrates, the songbird cerebrum has two halves (or hemispheres), each of which controls mainly the muscles in one half of the body. Many motor behaviors such as singing rely on high coordination of activity in both hemispheres, yet little is known about the neural mechanisms of this coordination. By using electrical stimuli to briefly perturb the activity of neurons in the motor pathway during song production, we study their involvement in generating the different elements of a song in zebra finches. We find mostly disjoint time intervals in which stimulation of either the right or left hemisphere is effective in distorting a song. This interhemispheric switching of stimulation effectiveness is evidence of a novel form of ping-pong–like motor coordination. Because left–right alternation is the basis of many motor patterns such as swimming and walking, we speculate that interhemispheric switching in songbirds has its evolutionary roots in older circuit principles invented for locomotion.

## Abstract 3

Motor dominance across cerebral hemispheres bounces back and forth like a virtual ping-pong ball during song production in zebra finches.

## Introduction

Owing to its complexity and high precision, birdsong has provided an important animal model for studies of motor control. Adult zebra finch songs are formed by repetitions of a highly stereotyped motif that is composed of two to eight syllables and is acquired from a tutor during a critical sensorimotor period [1]. Because the stereotypy of birdsong is sustained after removal of auditory feedback, birdsong has been thought to be organized by a “central motor program” [2–4].

The main cerebral brain areas for vocal production are the robust nucleus of the arcopallium (RA), HVC (used as a proper name), and the lateral magnocellular nucleus of the anterior nidopallium (LMAN), the latter of which forms the output of an avian basal-ganglia pathway [5]. Song-related neural activity in premotor brain areas is precisely coordinated across hemispheres, because both hemispheres contribute to the production of one unique and highly stereotyped song. This precise coordination is illustrated by the strong synchronization of multiunit activity in left and right HVC during singing [6].

**Figure 1:**
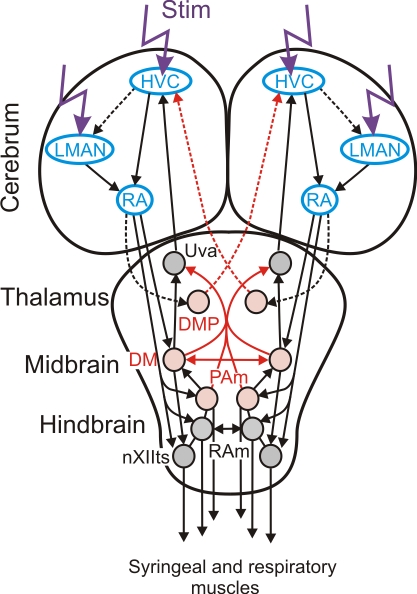
Schematic of Bilaterally Organized Song-Control Areas in the Zebra Finch Brain The output of the song system is formed by the tracheosyringeal portion of the hypoglossal nucleus (nXIIts), by inspiratory nucleus parambigualis (PAm), and by the expiratory nucleus retroambigualis (RAm), bottom. These motor areas receive input from cerebral song-control areas HVC and LMAN via RA (all shown in blue). There are two thalamic pathways for indirect interhemispheric communication between cerebral song-control areas: (1) via the nucleus uvaeformis (Uva) and (2) via the dorsomedial posterior nucleus (DMP). Areas with efferent projections to the contralateral hemisphere are marked in red, full lines show direct connections, and dashed lines show indirect connections. DM: dorsomedial nucleus of the intercollicular complex. Stimulation electrodes were implanted in HVC and LMAN (dark blue arrows).

A useful method to probe the functional roles of premotor brain areas is electrical stimulation. In general, electrical stimulation during motor production leads to specific behavioral distortions that depend on the location of stimulation electrodes [7,8] as well as on the stimulation time (or phase) within ongoing motor patterns [9–12]. For example, in LMAN, which is involved in modulating birdsong by social context [13,14], unilateral electrical stimulation induces small transient effects on sound amplitude or sound pitch, depending on the precise stimulation time within the ongoing song motif [10]. In HVC, which generates adult song by means of ultrasparsely firing “clockwork” neurons [15,16], unilateral electrical stimulation also leads to transient song degradations such as syllable distortions and syllable truncations [17]. More importantly, both LMAN and HVC stimulation sometimes induce nontransient effects such as song stoppings or early song restarts [4]. During such restarting events caused by HVC stimulation, ongoing premotor activity in the contralateral HVC is reset within a few tens of milliseconds [18]. Given that there are no direct interhemispheric connections between cerebral song-control areas, interhemispheric synchronization and resetting must rely on common inputs to the song-control system from interhemispherically connected mid- and hindbrain areas [19–23] (Figure 1).

To explore the mechanisms of interhemispheric coordination and the dependence of song distortions on stimulation time, we chronically implanted HVC in adult male zebra finches with bipolar stimulation electrodes. We trained an artificial neural network to reliably detect the earliest possible note in a song motif in real time and stimulated either right or left HVC with a brief 0.4-ms biphasic (0.2 ms/phase) current pulse at random time lags after detection. We frequently interleaved stimulation trials by catch trials in which no stimulation was delivered. We also explored temporally modulated effectiveness of LMAN stimulation by using suitable multipulse current trains delivered to bipolar stimulation electrodes implanted in LMAN [10].

## Results

**Figure 2:**
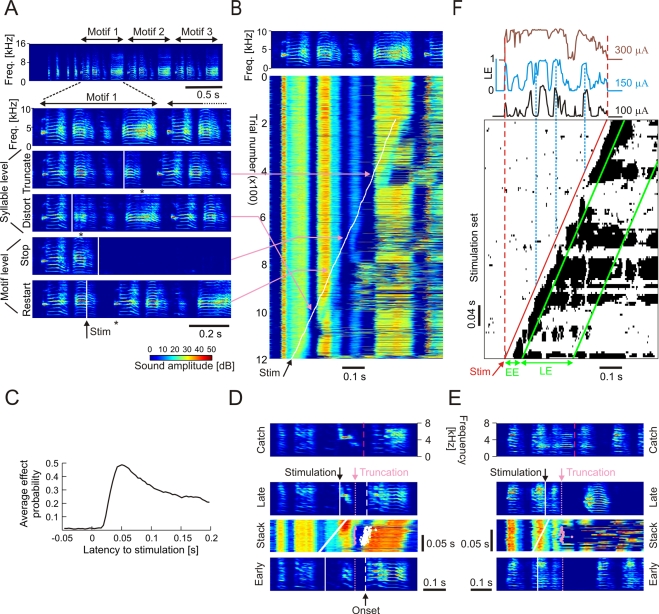
HVC Stimulation Leads to Song Distortions at the Syllable and the Motif Levels (A) Spectrogram of an adult male zebra finch song bout (top) with song motifs indicated by horizontal arrows. Below are a zoom into a normal (nonstimulated) song motif and examples of syllable-level stimulation (Stim) effects (truncation and distortion, indicated by asterisks), and examples of motif-level effects (stopping and restart, with the restarting point indicated by an asterisk). (B) Top: spectrogram of the song motif in (A). Bottom: sound-amplitude stack plot depicting stimulation effects observed over the course of a day. Trials are ordered by stimulation time, marked by white dots. The top 200 traces depict nonstimulated catch trials, revealing the high stereotypy of song motifs. The pink arrows indicate corresponding stimulation trials in (A). Stimulation effects display a contiguity, as revealed for example by nearby stimulation times that lead to persistent syllable truncations and early motif restarts (top pink arrow). (C) Effects on sound amplitudes started 20 ms after HVC stimulation and peaked after about 50 ms. Shown is the histogram of time bins with amplitude effects as function of latency to stimulation, normalized to the peak and averaged over stimulation sites (average over *n* = 20 HVC stimulation sites in 10 birds). On average, the peak effectiveness occurred after about 50 ms, well within the EE window. (D) An example in which HVC stimulation over a broad temporal range leads to identical syllable truncation times. In the unperturbed spectrogram (catch trial, top), we marked the normal offset time of a selected syllable with the magenta vertical dashed line. Below are song spectrograms with identical syllable truncation times (pink vertical dotted lines) for both early and late stimulation (stimulation times are marked by white vertical lines). The sound-amplitude stack plot (between the stimulation examples) reveals that all syllable truncations (pink dots, shown only for intermediate stimulation times) are vertically aligned, irrespective of stimulation time (white ramp dots, left). Note also that syllable truncations were followed by an unusual syllable that was never observed at this location during catch trials. The onsets of this appended syllable are marked by white vertical dashed lines in the “early” and “late” stimulation examples and by the cloud of white dots in the stack plot (shown for intermediate stimulation times). An *F*-test revealed that the onset times of this appended syllable had the same variance when measured relative to song detection time and relative to stimulation time (*p* = 0.1). Hence, the timing of this syllable was fixed neither to stimulation time nor to song time. (E) A stimulation example in a different bird in which syllable truncations (pink dots) tended to occur during a particular note rather than at a fixed time lag after stimulation. Same legend as in (D). (F) LEs increased with stimulation current. The LE curve associated with the stack plot in (B) is depicted by the blue curve (150 μA, single pulse). LE curves for higher and lower stimulation currents are also shown (brown and black curves). With increasing current, more stimulation times lead to LEs. The lower panel illustrates the computation of effect curves in this bird. The red line indicates stimulation time; black rasters indicate 3.9-ms time bins in which stimulation-related sound amplitudes were significantly different from baseline; and the green lines delimit the time windows in which EEs and LEs were read out. Note that the stack plot in (F) is not perfectly (horizontally) aligned with that in (B) because of randomness of stimulation times (wiggly white line in [B]).

In line with earlier work, we found that unilateral HVC and LMAN stimulation distorted songs at the levels of song syllables and song motifs (Figure 2A) [4,10,17,18]. By definition, syllable-level effects were restricted to the stimulated or the subsequent syllable and consisted of either syllable distortions or syllable truncations. On the other hand, motif-level effects were manifest in longer time windows after stimulation and consisted of sudden song stopping or early motif restarts (see Materials and Methods for exact definition of effects). The prevalence of syllable- and motif-level distortions caused by HVC and LMAN stimulation is reported for all birds in Table S1.

When hundreds of stimulated motifs were reordered by stimulation time, a temporal contiguity of stimulation effects became apparent in which nearby stimulation times led to qualitatively similar song distortions (Figure 2B). Hence, song distortions were not random, but were often deterministically linked with stimulation time, possibly caused by strong perturbation of stereotyped premotor activity.

A more detailed analysis revealed that song distortions most frequently occurred on the syllable level within several tens of milliseconds after stimulation. The probability of sound-amplitude distortions sharply increased 20 ms after stimulation, peaked roughly 50 ms after stimulation, and decayed thereafter (Figure 2C). This sharp rise agrees with measurements of air sac pressure deviations, the average onset of which lags HVC stimulation by 15–20 ms [17], whereas the late decay suggests that some perturbations of neural activity were transient and affected only a subpopulation of neurons.

Interestingly, on a fine time scale, not all distortions were locked to stimulation time. We occasionally observed syllables that were truncated, not with a fixed delay to stimulation, but during a fixed time point with respect to the unperturbed motif (Figure 2D and 2E). In these cases, stimulation needed to occur within some time interval before a particular note in order to truncate that note, revealing that the motor program exhibits time points of high perturbation sensitivity. And, more interestingly, sometimes stimulation effects such as early motif restarts occurred neither after a fixed latency to stimulation nor at a fixed time point of the unperturbed motif, but at some intermediate time (Figure 2D), further demonstrating nonlinear timing aspects of the song motor program.

We automated the inspection of song distortions by analysis of sound amplitudes. We were mostly interested in motif-level effects because these seemed to arise from wide-spread and irreversible perturbation of premotor activity. For each stimulation time, we computed a late-effect (LE) value, defined as the fraction of 3.9-ms time bins in a 78–312-ms window after stimulation in which sound amplitudes were significantly different from amplitudes recorded during catch trials (see Materials and Methods). LE curves as a function of stimulation time had many sharp peaks that corresponded to different motif-level effects, separated by troughs in which stimulation was rather ineffective (on the motif level). When we increased the stimulation currents, the set of effective stimulation times grew, as revealed by LE peaks that grew in height and width (Figure 2F). At the extreme of very high currents on the order of 0.5–1 mA, birds always stopped singing, and significant LEs were seen for all stimulation times (*n* = 3 birds, unpublished data). In this study, our experimental strategy was to rapidly tune stimulation currents in order to observe highly modulated LE curves with coexistence of very large and close to zero values, a task that typically was achieved within 2 d. At the current intensities chosen, LE curves displayed diverse peaks (the mean peak width at the effectiveness threshold was 20 ms, median 8 ms, range 4 to 160 ms, *n* = 20 HVC stimulation sites in 10 birds). This wide range of peak widths in LE curves indicates that HVC stimulation perturbed neural activity on multiple time scales. The strong modulation of LE curves suggests rapid waxing and waning of the ipsilateral HVC drive, raising the question about modulation in the contralateral hemisphere.

**Figure 3:**
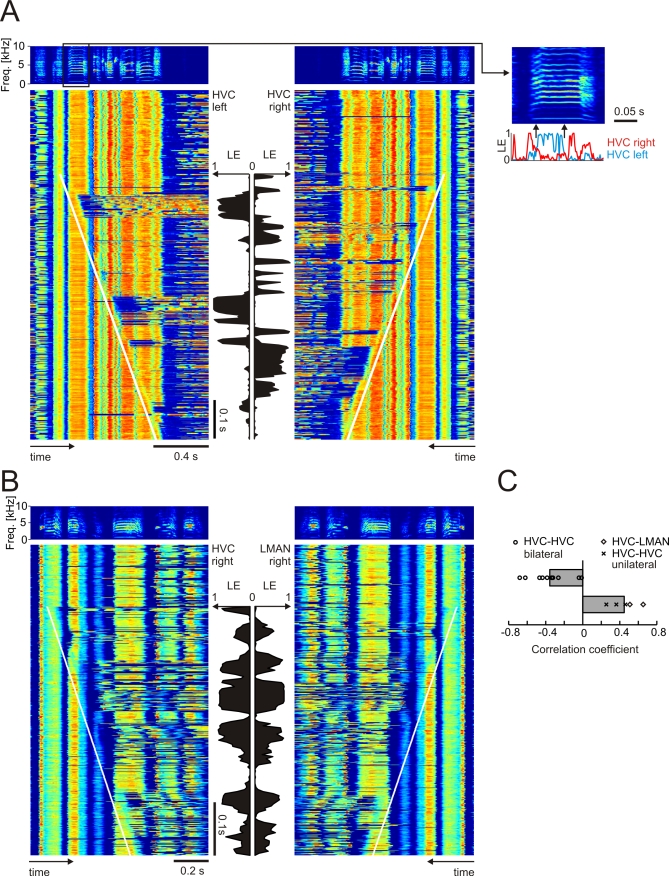
Interhemispheric Switching of Stimulation Effectiveness (A) Across hemispheres, effective HVC stimulation times are complementary. Shown are sound-amplitude stack plots for left HVC stimulation (left) and for right HVC stimulation (right). Corresponding LE curves (filled) are plotted against each other in the center to illustrate the alternating stimulation effectiveness. The CC between left and right LE curves in this bird is −0.62. Inset (long black arrow): a zoom into the harmonic stack reveals no obvious relation between spectral syllable composition and right/left LE curves shown below: The transition from left to right effectiveness (right arrow) occurs in the middle of the harmonic stack where there is no apparent song change. (B) Within hemispheres, effective stimulation times are strongly overlapping. Shown are sound-amplitude stack plots for HVC stimulation in right hemisphere (HVC-R) and for ipsilateral LMAN stimulation (LMAN-R). Corresponding LE curves (filled) are plotted against each other in the center to illustrate the alternating stimulation effectiveness. The CC between left and right LE curves in this bird is 0.65. (C) Bar plot, showing the average CC of LE curves for stimulation in opposite hemispheres (top) and in the same hemispheres (bottom). Symbols indicate CCs in individual birds: for unilateral HVC-HVC stimulation using two electrode pairs (crosses), for unilateral HVC-LMAN stimulation (diamonds), and for bilateral HVC-HVC stimulation (circles).

To probe evidence of lateralized stimulation effectiveness, we implanted birds with stimulation electrodes in both left and right HVC, and performed unilateral stimulation in randomly chosen hemispheres and at random time lags after note detection. After sorting all trials recorded over 1–3 d by hemisphere and stimulation time, a remarkable complementarity became apparent: For most stimulation times, stimulation effects were seen either for right- or left-side stimulation, but not for both (Figure 3A; see Figure S1 for all birds used in our study). LE curves associated with left and right HVC stimulation were strongly modulated, but in an alternating fashion. We quantified the interhemispheric complementarity of stimulation effectiveness by the correlation coefficient (CC; see Materials and Methods) between right and left LE curves, and found that negative correlations prevailed (average CC −0.36, range −0.68 to −0.01, *n* = 10 birds). To assess the significance of these anticorrelations, in three birds we implanted two pairs of stimulation electrodes in right HVC (in a cross arrangement). By running the same experimental protocol on the two ipsilateral stimulation sites in HVC, we found that CCs between corresponding LE curves were positive (average CC 0.36, range 0.25 to 0.46, *n* = 3 birds), illustrating that the dependence of stimulation effects on electrode position within HVC is weak and demonstrating that the anticorrelation of stimulation effectiveness in bilateral stimulation experiments was highly significant. Moreover, in two birds, we implanted stimulation electrodes in right HVC and right LMAN, and also found positive CCs between corresponding LE curves (0.65 and 0.51, Figure 3B). The CCs in all birds are depicted in Figure 3C (see Table S2 for additional characterizations of the complementarity of right and left LE curves). We interpret this complementarity as evidence that interhemispheric motor coordination involves temporally alternating neural mechanisms.

We were interested in determining whether the events at which stimulation effectiveness switched from one hemisphere to the other were locked to salient song features and whether the resulting switching intervals obeyed any regularity. Visually, the effectiveness of electrical stimuli appeared to switch several times from one hemisphere to the other within a song motif, but often there was no obvious relationship between the discrete switching events and the sound spectrum produced at these times (inset of Figure 3A). When we assessed the events at which the effectiveness of electrical stimuli switched from one hemisphere to the other in terms of onsets of contralateral effectiveness (LE values larger than baseline), the mean switching interval was 35 ms (median 28 ms, range 4 to 150 ms). By contrast, when the switching events were defined by joint occurrence of ipsilateral ineffectiveness and onsets of contralateral effectiveness, the mean interval was 64 ms (median 44 ms, range 4 to 240 ms). Hence, on average, stimulus effectiveness switched back and forth between hemispheres within a few tens of milliseconds. However, our estimates of lateralized effectiveness and switching intervals must be interpreted with caution because of the aforementioned dependence of LE peak widths on stimulus current, implying that switching intervals depend (nontrivially) on stimulus current. Nevertheless, because we found broadly distributed switching intervals both across all birds and within single birds, there is little evidence of periodicity in this interhemispheric switching process.

We further explored whether effective stimuli and their lateralization were related to specific sound features. Zebra finches mostly expire during syllables and inspire during syllable gaps [24]. Both expiratory and inspiratory nuclei in the brainstem project bilaterally and therefore may be involved in controlling effectiveness switching. Because we did not measure bronchial air flow, here we inferred respiratory patterns from sound pitch curves using the simplifying assumption that zero pitch during syllable gaps corresponded to inspiration and nonzero pitch to expiration. We defined a rhythm curve as being equal to one during expiration and zero during inspiration. There was no significant coherence between this rhythm curve and either the right or left LE curves (see Materials and Methods). These results were unchanged when we defined expiratory patterns in terms of pitch values in the limited range 20–5,000 Hz (thereby assuming that some high-pitched notes are generated during inspiration). Similarly, there was no significant coherence between right/left LE curves and each of the following: sound-amplitude curves, pitch curves (see Materials and Methods), syllable onset curves, and syllable offset curves (the latter were binary curves in which a pulse of variable width was set at the transitions between inspiration and expiration as assessed by the rhythm curve). Thus, the evidence for a consistent relationship between stimulation effectiveness and simple sound features is rather weak. Notice though that all our conclusions were reached from just a few seconds of effect-curve data (15 birds) and that it would be worthwhile to reinvestigate the relation between stimulation effectiveness and song features in the future provided a larger body of interhemispheric stimulation data will be available.

## Discussion

We have demonstrated an interhemispheric switching process for vocal production. In this process, the motor program exhibits perturbation sensitivity that rapidly alternates between hemispheres. Such alternation is surprising given that HVC activity is highly synchronized across hemispheres during singing, and suggests that motor dominance rapidly switches back and forth between hemispheres. Possibly, the apparent alternation of dominance is related to birds' ability to independently control the two halves of their vocal organ [25,26]. However, alternation is not synonymous with independent control as it represents a restriction on independence. It is difficult to ascertain which hemisphere is dominating at any time in this switching process, because we were not able to find a simple relationship in zebra finches between stimulation effectiveness and either song features or song rhythm. On the one hand, one could argue that stimulation should be more effective in a dominant hemisphere because this hemisphere is being perturbed while generating a song in both syringeal halves. On the other hand, one could argue that stimulation should be less effective in a dominant hemisphere because the perturbation is not strong enough to overrule the ongoing activity there. In the following, we discuss the evidence for these two interpretations, as well as for interpretations on whether stimulations perturb activity in local or in distributed networks.

From existing data, we cannot infer whether or not the motor apparatus necessitates continuous and simultaneous drive from both cerebral hemispheres: adult birds do not sing normally after unilateral RA lesions [19], but these data do not exclude the possibility that at any time, the effective motor program resides in just a single hemisphere and bounces back and forth between hemispheres during singing. For example, if singing at all times is based on activity in just a single hemisphere and the drive provided by premotor activity in the other hemisphere is temporarily gated off, then we would conclude that the dominant hemisphere is the one in which low-intensity stimulation is effective. In this view, stimulation of the nondominant hemisphere above a given current threshold would also be able to distort songs, because strong perturbations might ultimately find their way to the dominant side (past the gate) where they could interrupt the ongoing motor program. However, if normal song production at all times requires simultaneous contributions from both hemispheres, then high stimulation effectiveness might be an attribute of the nondominant hemisphere, because this hemisphere can be perturbed at lower stimulation currents. On the dominant side then, low-intensity stimulation would be corrected by redundant neural mechanisms that were not sufficiently perturbed by the stimulation.

Not only the dominance question is difficult to address, but it is similarly difficult to tell whether song disruptions were entirely due to perturbation of local ongoing HVC activity or of a larger distributed network. For example, the number of spiking RA-projecting HVC neurons might drift randomly up and down during the song motif (with some inertia). Such random drifts could be associated with a compensatory increase in the number of spiking neurons in the contralateral HVC and thus to alternation of dominance. A compensatory process could be regulated during song development (e.g., by neurogenesis [27] and programmed cell death), and therefore alternating dominance would not have to rely on real-time interhemispheric communication. According to this interpretation, LMAN and ipsilateral HVC stimulation lead to similar song distortions because LMAN stimulation perturbs RA-projecting HVC neurons, for example, via RA [28]. Although at this stage we cannot rule out this scenario, it is unclear why compensatory mechanisms would act across hemispheres, but not within the same hemisphere. Furthermore, it is difficult to reconcile this scenario with observations of interhemispheric synchronization of HVC activity and with some stimulation effects such as early song restarts.

The more likely scenario within which our observations can be explained is that LMAN and HVC stimulation induce similar song distortions because of widespread perturbation of subpallial structures via RA. Because we observed a wide range of switching intervals, we found little support for the idea that switching times are determined by fixed signal propagation times (for example as reverberating activity in closed synaptic loops) or by the fixed period of a simple pattern generator circuit. Rather, some switching events may arise from detection of specific premotor patterns in one hemisphere that are subsequently relayed to the contralateral hemisphere.

Interhemispheric switching processes in relation to motor production have been reported also in mammals, for example during the preparation of vocal production in humans, in which effectiveness of transcranial magnetic stimulation (TMS) of motor cortex alternates between hemispheres [12]. Interhemispheric switching has also recently been shown to exist during perceptual rivalry, as evidenced by the hemispheric dependence of magnetic and calorimetric stimulation [29]. Interhemispheric switching may thus be a fundamental mechanism by which sensory and motor-related activity is coordinated across hemispheres. In mammals, interhemispheric coordination seems to be mainly mediated by corticocortical projections [30,31]. However, during saccadic eye movements of split-brain monkeys, activity in the two hemispheres has been shown to remain coordinated despite the lack of cerebral commissures, suggesting that subcortical pathways can subserve coordination also in the mammalian brain [32], and suggesting that similarities may exist between interhemispheric coordination in avian and mammalian brains.

Based on networks models, switching has been proposed to depend on competitive interactions [33] mediated by inhibition [34]. Evidence for interhemispheric inhibition has been found in TMS studies of human motor cortex [35,36]. We speculate that interhemispheric switching in songbirds could also rely on inhibitory mechanisms. A possible function of such inhibition could be to suppress mirror-symmetrical movements, which are thought to represent one of the default operation modes of bilateral motor systems [37]. In this sense, interhemispheric inhibition would coexist with more cooperative (excitatory) interactions between hemispheres. Inhibitory gating mechanisms could be mediated, for example, via tonically spiking Uva projection neurons (see also Figure 1) [38], and excitatory mechanisms could be relayed by respiratory nuclei, known to generate mirror-symmetrical respiratory patterns [39].

The reported interhemispheric switching process is reminiscent of one of the most prominent motor programs with left–right alternating dynamics, which is locomotion. In vertebrates, locomotion is subserved by central pattern generators in the spinal cord, which can display sustained rhythmic activity with left–right alterations even in in vitro preparations [40]. Because locomotion is much older than birdsong on an evolutionary time scale, phase-alternating neural circuits must have existed long before birds started to sing. Possibly, principles of limb coordination in locomotor circuits have been replicated by evolution for the more recent advent of birdsong. Some support for this idea comes from the conservation of bilateral projection patterns in brainstem nuclei of songbirds and non-songbirds [41], suggesting that old brain circuits have evolved to support new functions.

## Materials and Methods

### Subjects.

Adult (>90 d old) male zebra finches (Taeniopygia guttata) were used for experiments. Birds were selected on the basis of singing frequency and song complexity, and were isolated in a sound-attenuating chamber. To maximize singing frequency, birds had visual contact to one or more female zebra finches through the glass door of the chamber. A total of 15 birds were used; data in one bird were discarded because HVC stimulation did not reliably produce motif-level effects. At the end of experiments, electrolytic lesions were performed at the stimulation sites by DC current injections (15 μA for 20 s), birds were killed by overdose of Nembutal, and stimulation sites were verified in histological brain sections. All procedures were approved by the Veterinary Office of the Kanton of Zurich.

### Song-triggered microstimulation.

We delivered electrical stimuli with uniformly distributed probability over the time span of song motifs using custom written Labview software (National Instruments Corporation). With probability 0.35, detection triggered microstimulation at site A, with probability 0.35 at site B; and with probability 0.3, no stimulation was delivered (catch trials).

Electrodes were made of 50-μm stainless steel wire. Electrical stimuli in HVC consisted of a single 0.4-ms biphasic (0.2 ms/phase) current pulse of amplitude between 100 μA and 1 mA. In LMAN, electrical stimuli consisted of trains of ten biphasic current pulses at 400 Hz (0.4 ms/phase; train duration 23.3 ms) and amplitudes in the range 10–100 μA. The current threshold at which single-pulse stimulation in LMAN induced motif-level effects (song suspensions) was high (typically >1 mA). For this reason and to adhere to previous stimulation studies [4,10], we chose a multipulse paradigm in LMAN in which we stimulated for ten pulses at low currents (10 ∼ 100 μA per pulse).

### Analysis of syllable- and motif-level effects.

We distinguished among different syllable and motif-level effects as follows:

*Syllable truncations.* First, we measured baseline distributions of syllable lengths from data of selected catch trials (only complete motifs). Stimulated syllables were then classified as truncated if their duration was within the lowest percentile of the baseline distribution. We searched for truncations only in a time window up to 156 ms (corresponding to 40 time bins of 3.9 ms or 128 sound samples each) after stimulation.

*Syllable distortions.* In each time bin after note detection, we calculated the baseline distribution of sound amplitudes during selected catch trials (no spontaneous song stopping). We then counted the number of 3.9-ms time bins up to 78 ms post stimulation time in which the stimulation-related sound amplitudes were significantly different from baseline (percentile *p* < 0.025 or *p* > 0.975). If this number was large enough (binomial test, alpha = 0.05), then we classified this stimulation effect as a syllable distortion. Distortions and truncations were not mutually exclusive.

*Motif stoppings and restarts.* For each bird, we chose a sound-amplitude threshold slightly above cage-noise level (we found that a threshold of 20% into the 1–99th percentile interval worked well for all birds). For all stimulation trials, under visual supervision, we then used this threshold to mark the offset time of every prematurely stopped motif and the successive restart time of the following note (independently of whether this note come from a song syllable, an introductory note, or a call). If the offset time fell into a window from 0 to 156 ms after stimulation and there was no restart until 312 ms, we then classified the stimulation effect as a stopping event. If, on the other hand, there was a restart after a premature offset within 312 ms after stimulation, then the stimulation effect was a restart. Hence, restarts and stoppings were mutually exclusive (however, song stoppings and syllable truncations were not).

### Automated analysis of stimulation effects in early- and late-effect windows.

All songs (stimulation and catch trials) were aligned by detection time. For each stimulation site, we sorted the trials by stimulation time and grouped them into 9.75-ms sets with centers separated by 3.9 ms from each other. With a mean stimulation range of approximately 500 ms and typical detection of 800–2,000 song motifs per day, we obtained roughly three to eight stimulation trials per set per day. Typically, we collected a mean of 10–20 trials per set and then tested for each set whether the sound amplitudes in 3.9-ms bins after stimulation were different from amplitudes in matched time bins during catch trials using the Kolmogorov-Smirnov (KS) test (*p* < 0.01). For each set, we quantified the stimulation effect by the fraction of time bins in which significant differences were detected. LE curves were based on bins ranging from 78 to 312 ms after stimulation (bins 21 to 80). Early-effect (EE) curves were based on bins ranging from 0 to 78 ms after stimulation (bins 1 to 20). To assess the time scales of song perturbations, we computed the peak widths in LE curves at the effectiveness threshold, defined by the baseline LE value during catch trials (binomial test, *p* < 0.01).

### Notes on robustness.

Our results did not depend critically on the EE and LE time windows in which syllable-level and motif-level effects were assessed. We chose the offset of the LE window (312 ms) as a compromise between being large enough to yield high sensitivity and small enough to not extend too far beyond the motif end where songs became highly variable. We set the onset of the LE window (or offset of the EE window, 78 ms) so as to exceed the peak time of stimulation effectiveness (Figure 2D), which was within 70 ms of stimulation (in agreement with previous reports [4]). Small changes in the LE window onsets (from 58.5 to 117 ms) and LE window offsets (from 234 to 390 ms) did not affect our findings of interhemispheric switching in any way.

By experimental design, our results were robust to variability in sound amplitudes caused by movements of the bird's head relative to the microphone. That is, head-position variability must have had identical influences on sound amplitudes recorded during catch trials and during stimulation trials because we randomly chose all stimulation parameters right after each detection event (i.e., whether and where to stimulate, and the stimulation time). Hence, by design there were no correlations between head position and stimulation parameters.

### Similarity of effect curves.

We assessed the similarity between effect curves *x* and *y* associated with different stimulation sites by the (Pearson) CC: 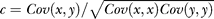 , where *Cov*(*x*,*y*) is the covariance between *x* and *y*. Because effect curves were nonnegative, stimulation times for which both *x* and *y* were ineffective (compared to sound-amplitude variability before stimulation, binomial test at 99% significance level) imposed a bias toward positive correlations. To avoid this bias, we ignored bilaterally ineffective stimulation times when calculating the CC (for LE curves, these were 32% of all stimulation times). Note that our conclusions were unchanged when CCs were calculated over the full set of stimulation times (thereby imposing a positive bias): the difference between average CCs in unilateral and bilateral stimulation experiments was highly significant in either case (*p* < 0.001, Wilcoxon rank sum test).

### Respiratory rhythm inferred from sound pitch.

The relationship between right/left LE curves *y* and the rhythm curve *z* was investigated by the coherence 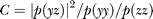 , where *p*(*yz*) is the cross-spectral density, and *p*(*yy*) and *p*(*zz*) are the power spectral densities of LE and rhythm curves, respectively. We chose the coherence function because its phase insensitivity allowed us to detect significant correlations irrespective of their time lag. We assessed the significance of coherence peaks by testing whether these exceeded two jackknife estimates of standard deviation (corresponding to 95% confidence). The ten jackknifes were defined by leaving out each of the ten birds from the analysis.

### Early-effect analysis.

By visual inspection, stimulation effectiveness at the syllable level showed weaker interhemispheric complementarity than effectiveness at the motif-level. Yet, EE curves associated with bilateral stimulation (average CC −0.13, range −0.68 to 0.43, *n* = 10) showed significantly lower correlations (*p* = 0.019, Wilcoxon rank sum test) than EE curves associated with unilateral stimulation (average CC 0.35, range 0.04 to 0.80, *n* = 5 birds). As before, to compute these CCs, we only considered stimulation times that were associated with effectiveness in at least one hemisphere, thereby omitting 25% of stimulation times (compared to omission of 32% for LE curves). In conclusion, alternating effectiveness was seen most clearly for stimuli that disrupted normal singing, but also for stimuli that induced immediate amplitude distortions.

### Pitch analysis.

We investigated the possibility that pitch differences exist between times at which right and left HVC stimulation is effective. The coherence between the sound pitch curve and either right or left LE curve was not significant, neither when we considered the full pitch curve nor when we clamped the pitch curve to zero below either 2 or 5 kHz. Similarly, the median pitch during right-effective stimulation was not statistically different from the median pitch during left-effective stimulation (Wilcoxon rank sum test, *p* = 0.4). We also tested whether pitch differences were seen at a particular time lag after effective stimulation times. We found that the median pitch 40 ms after left-effective stimulation was significantly higher than 40 ms after right-effective stimulation (*p* = 0.031, *n* = 10 birds). However, when we excluded any one of two particular birds from the analysis, then significance broke down (*p* > 0.1). Significance also broke down when assessed using a shuffle predictor of pitch differences in songs of randomly shuffled syllables and gaps from different birds (Monte Carlo simulations, *p* > 0.05).

## Supporting Information

Figure S1 Sound-Amplitude Stack Plots along with the Corresponding LE Curves (as in Figure 3A and 3B) for All Birds Used in Our Study (3.08 MB PDF)

Table S1 Percentages of HVC and LMAN Stimulations That Lead to Syllable-Level Effects (Truncations and Distortions) and to Motif-Level Effects (Stoppings and Restarts) R and L refer to the right and left hemisphere, respectively; Short and Long refer to the interelectrode distance in HVC-implanted birds; and the row labeled StDev contains the standard deviations across birds.
(58 KB DOC)

Table S2 Complementarity of Stimulation Effectiveness in Two Hemispheres Reported are the percentages of stimulation times that were associated with significant late effects (LEs) on sound amplitudes (first two columns). Also reported are the percentages of stimulation times associated with overlapping/nonoverlapping LEs at the two stimulation sites (third- and second-last columns), and the CCs of the associated LE curves (last column). Percentages for each bird are indicated relative to the set of all stimulation times. Labels are as defined in the legend of Table S1.
(49 KB DOC)

## Abbreviations

CC: correlation coefficient
EE: early effect
LE: late effect
LMAN: lateral magnocellular nucleus of the anterior nidopallium
RA: robust nucleus of the arcopallium
