# Supplementary material for: Rapid Interhemispheric Switching during Vocal Production in a Songbird
Source: PLoS Biol. 2008 Oct 14;6(10):1–9. doi: 10.1371/journal.pbio.0060250 (PMC2567002; doi:10.1371/journal.pbio.0060250)
Supplement: Supplementary file 1 [file pbio.0060250.sg001.pdf]

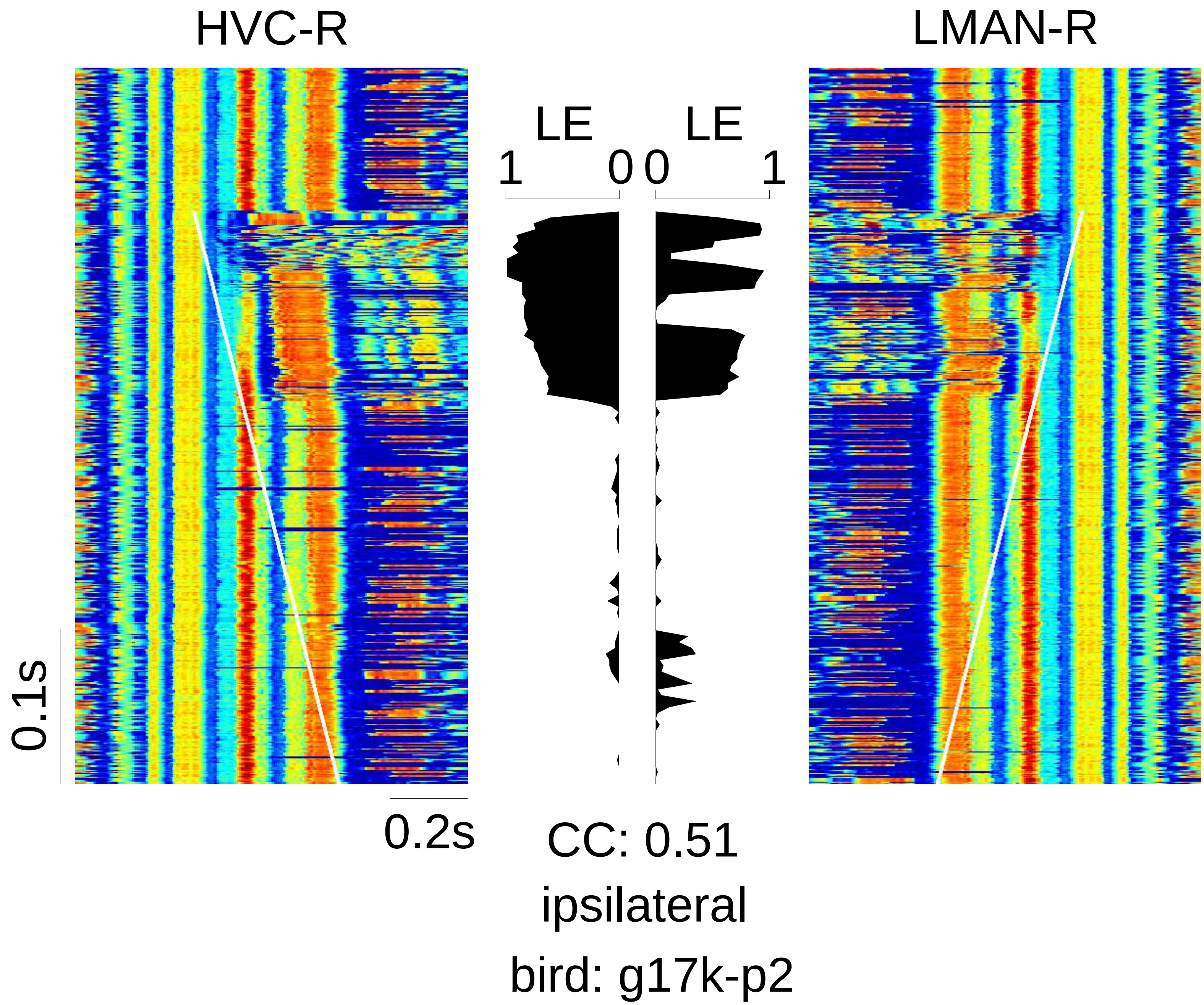
HVC-R
LMAN-R
LE
LE
1
0
0
1
0.1s
0.2s
CC: 0.51
ipsilateral
bird: g17k-p2

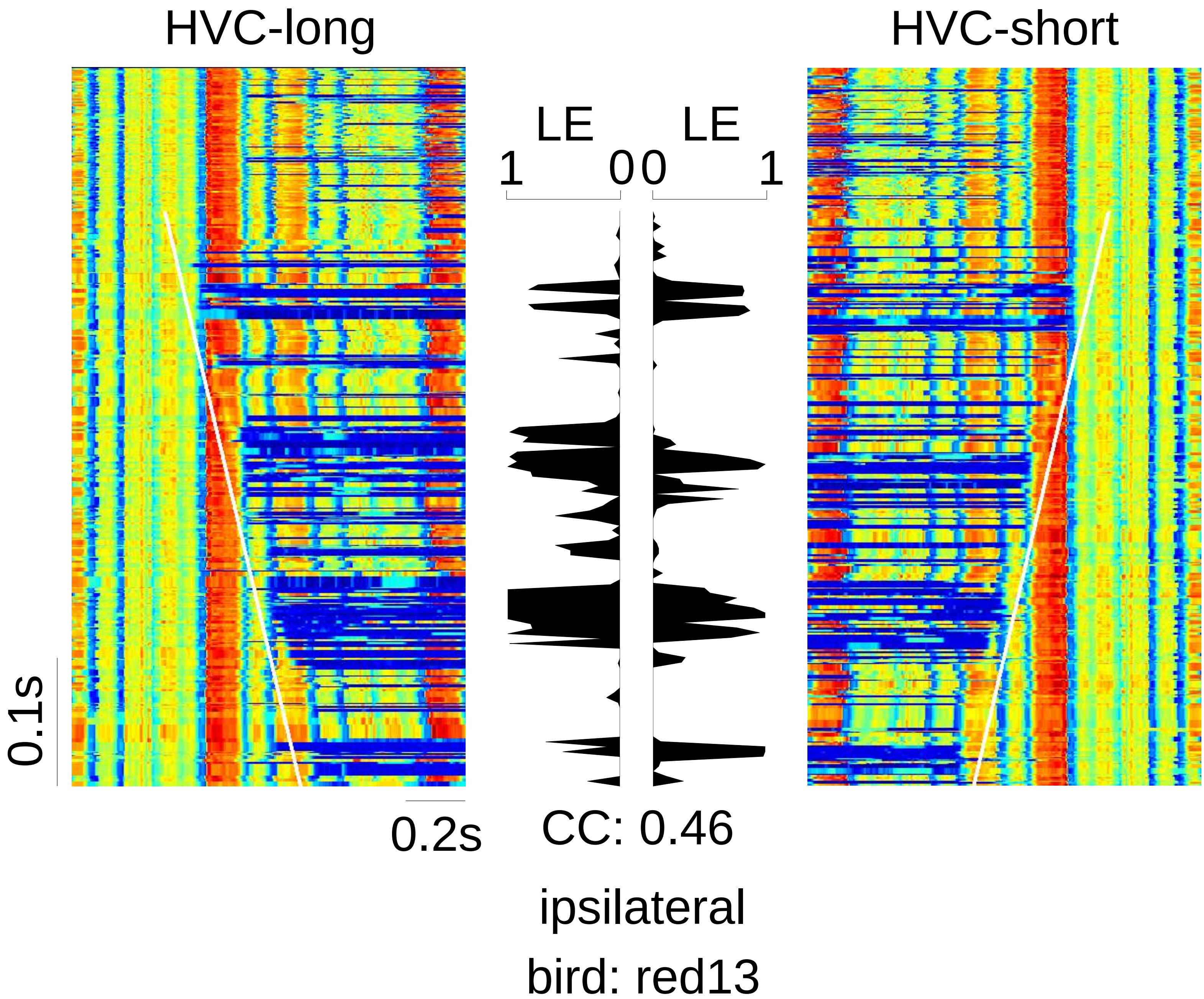
HVC-long
HVC-short
LE
LE
1
0
0
1
0.1s
0.2s
CC: 0.46
ipsilateral
bird: red13

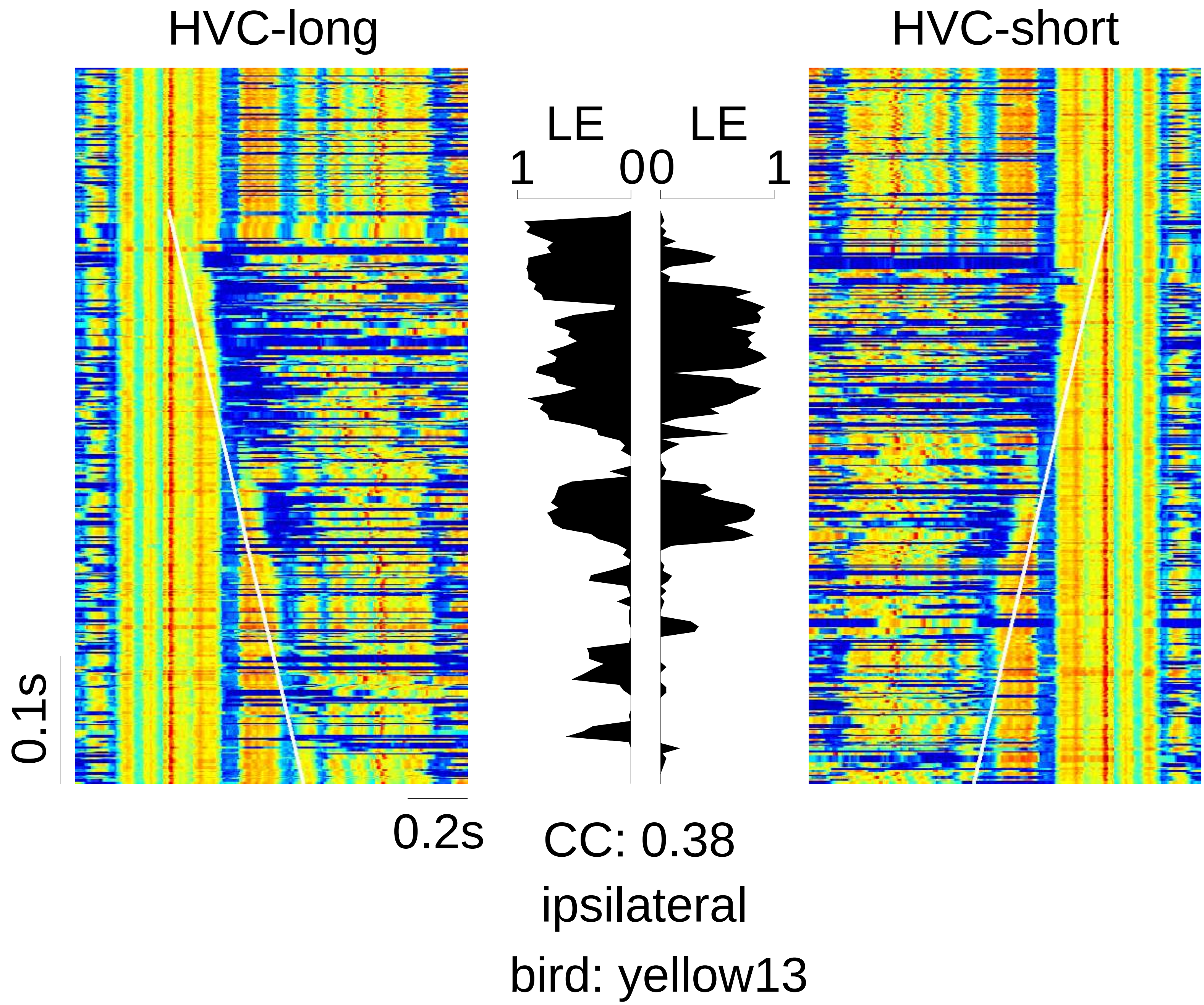
HVC-long
HVC-short
LE
LE
1
0
0
1
0.1s
0.2s
CC: 0.38
ipsilateral
bird: yellow13

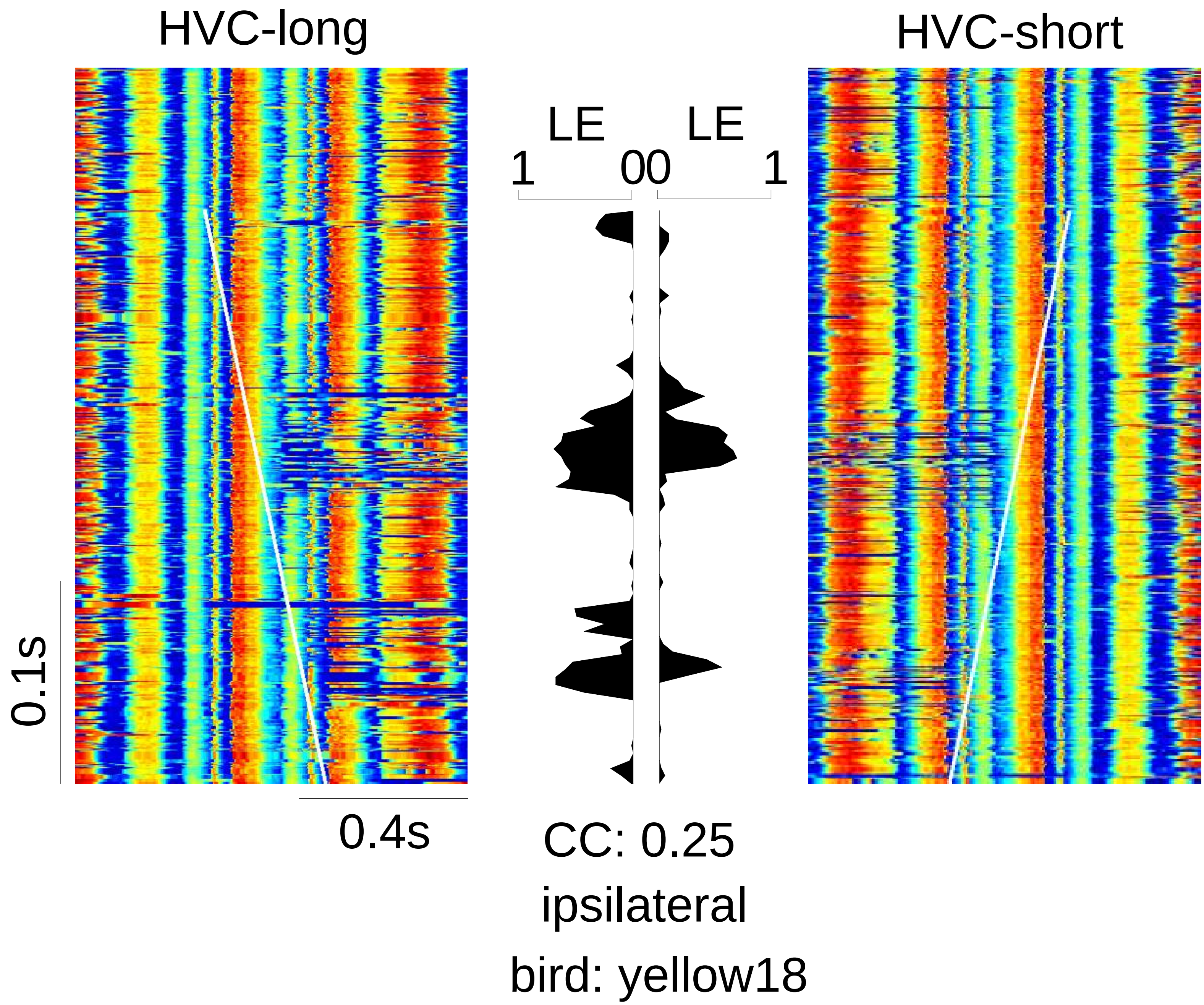
HVC-long
HVC-short
LE
LE
1
00
1
0.1s
0.4s
CC: 0.25
ipsilateral
bird: yellow18

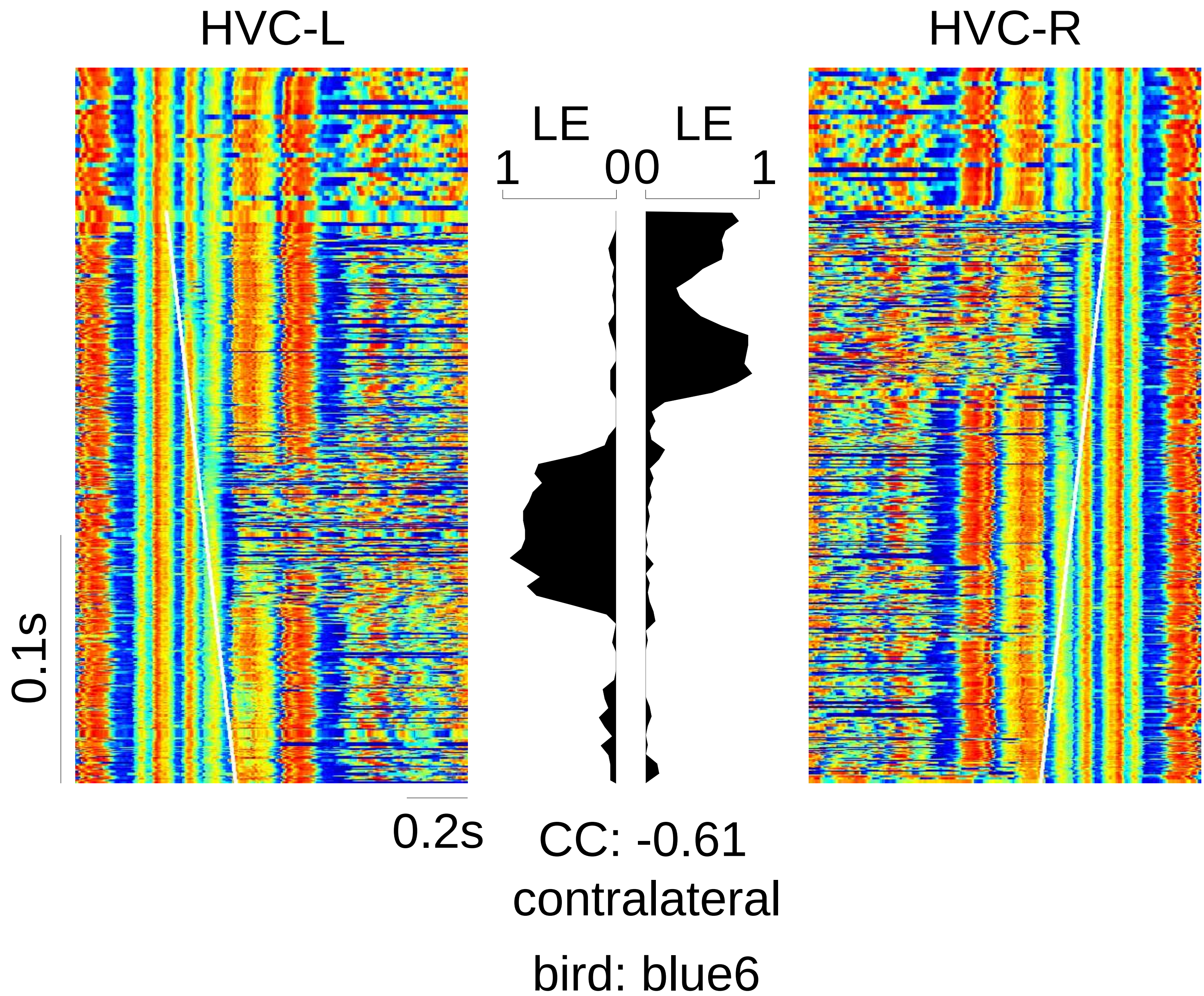
HVC-L
HVC-R
LE
LE
1
00
1
0.1s
0.2s
CC: -0.61
contralateral
bird: blue6

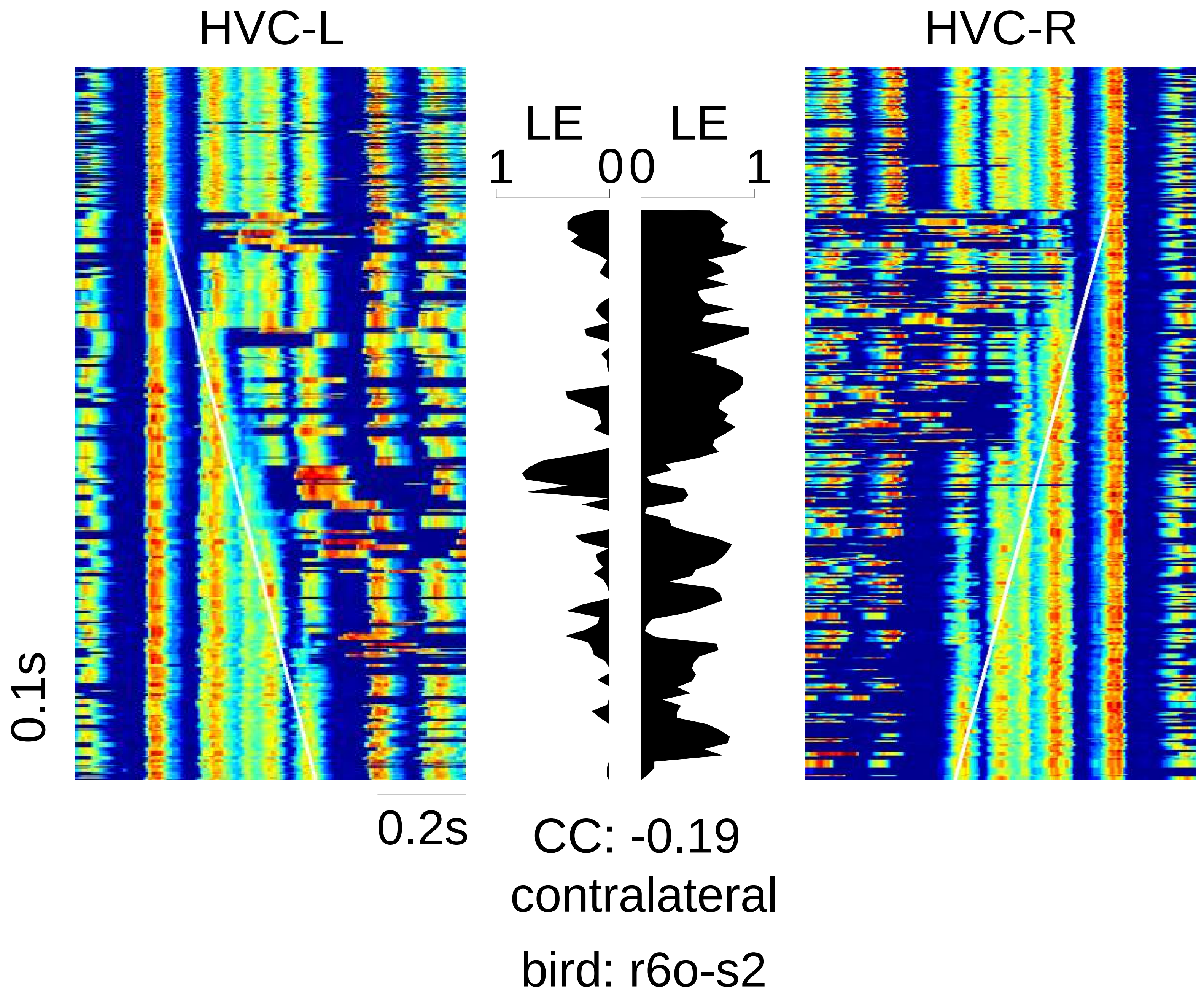
HVC-L
HVC-R
LE
LE
1
0
0
1
0.1s
0.2s
CC: -0.19
contralateral
bird: r6o-s2

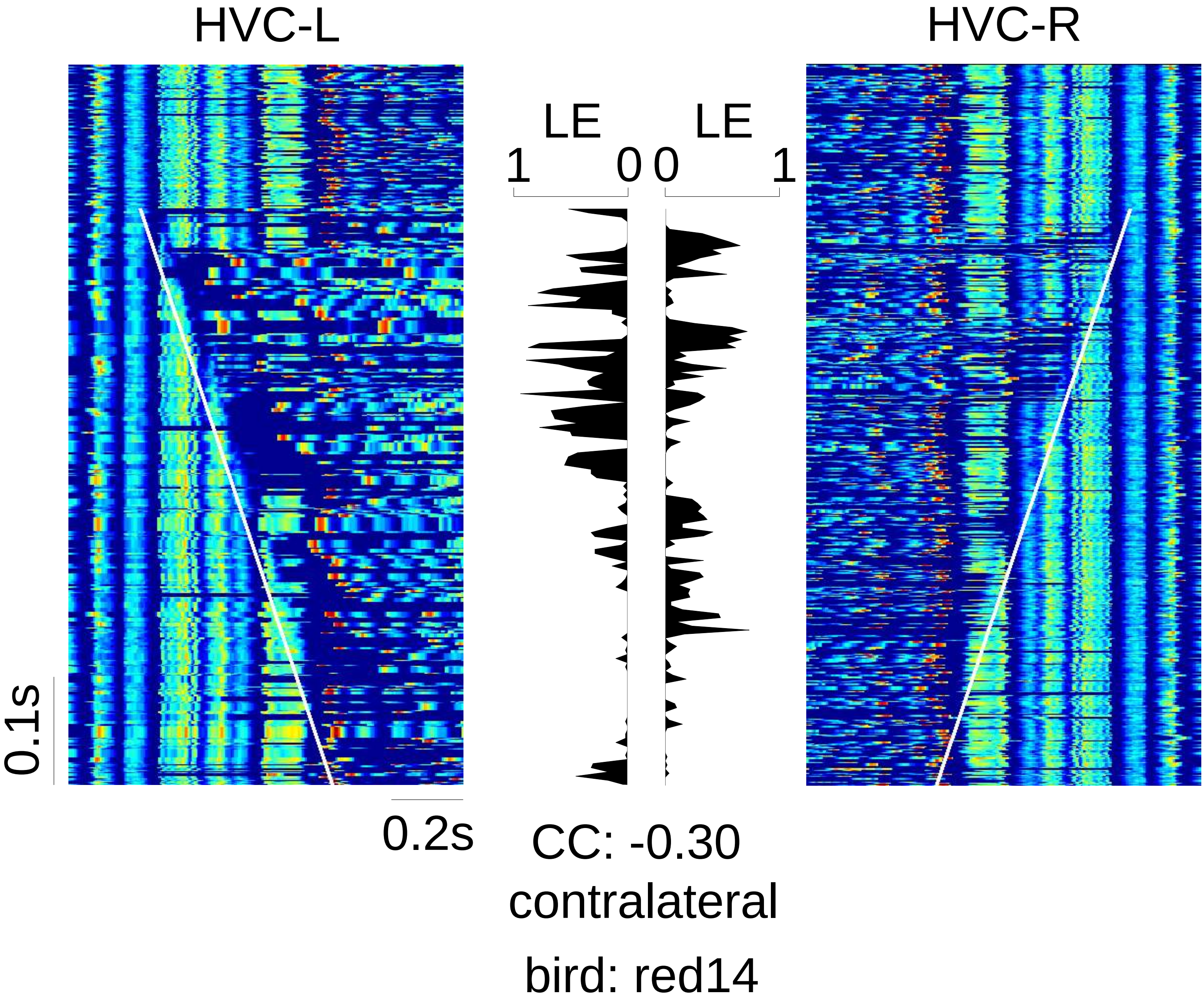
HVC-L
HVC-R
LE
LE
1
0
0
1
0.1s
0.2s
CC: -0.30
contralateral
bird: red14

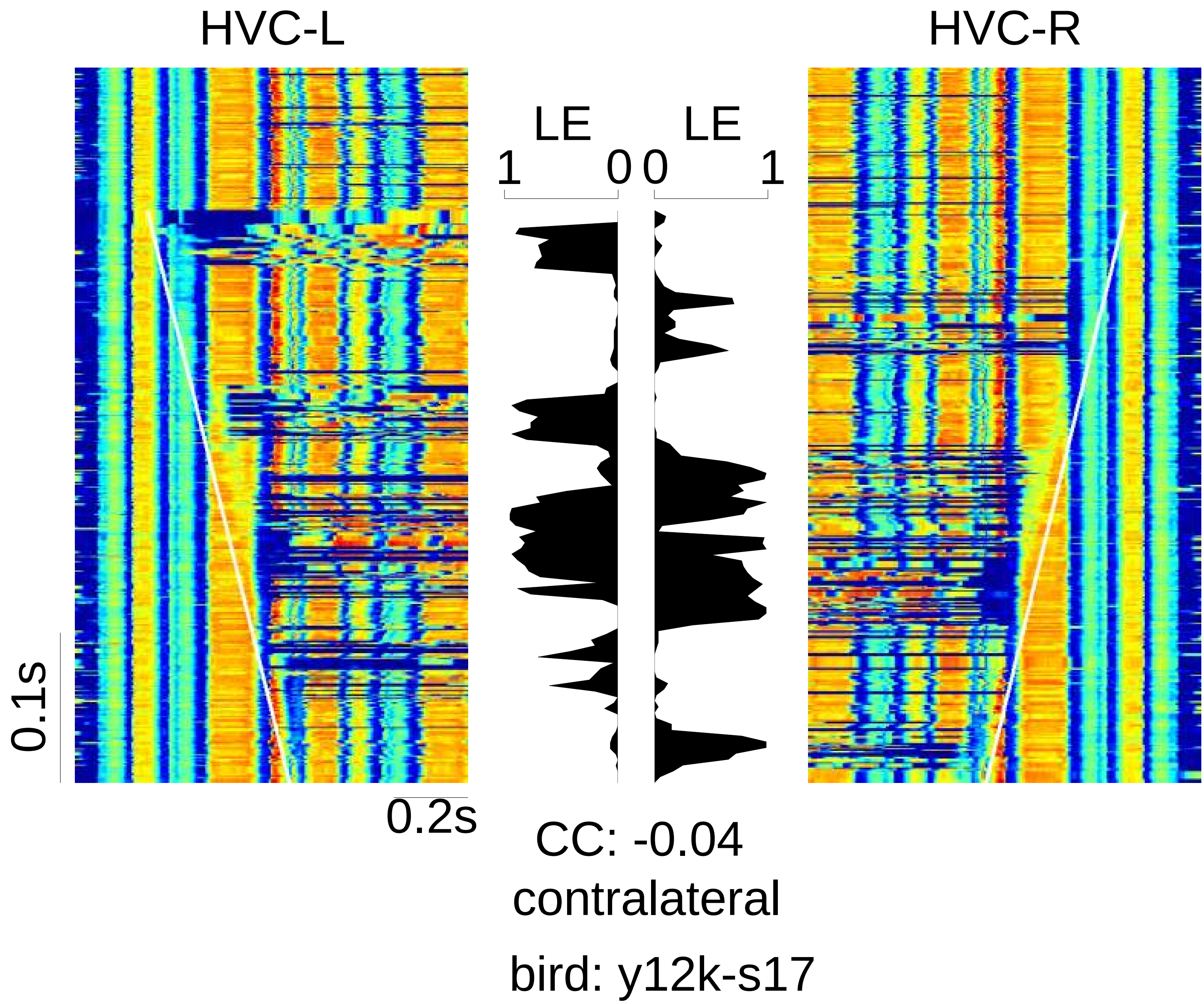

HVC-L
HVC-R
LE
LE
1
0
0
1
0.1s
0.2s
CC: -0.04
contralateral
bird: y12k-s17

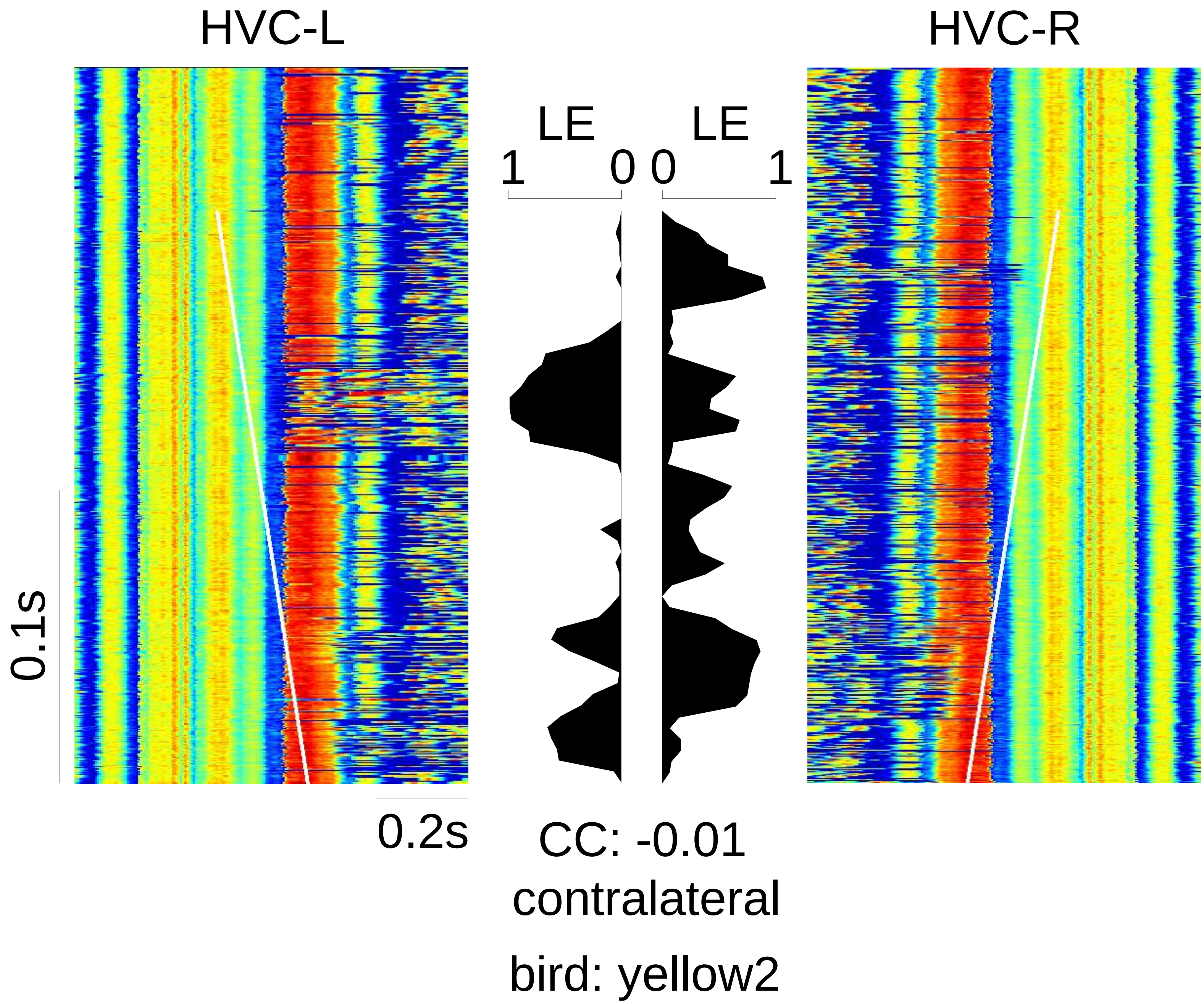
HVC-L
HVC-R
LE
LE
1
0
0
1
0.1s
0.2s
CC: -0.01
contralateral
bird: yellow2

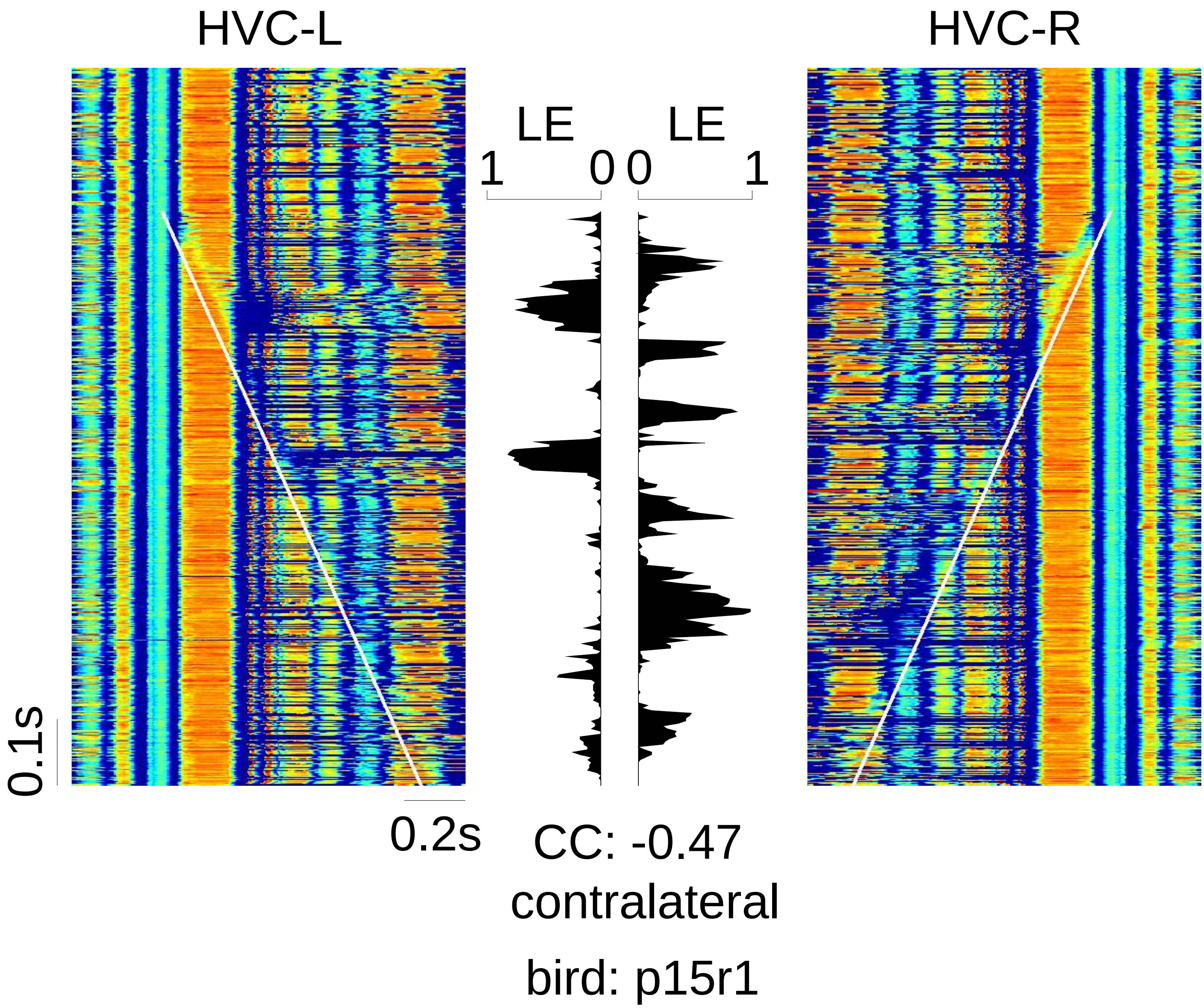

HVC-L
HVC-R
LE
LE
1
0
0
1
0.1s
0.2s
CC: -0.47
contralateral
bird: p15r1

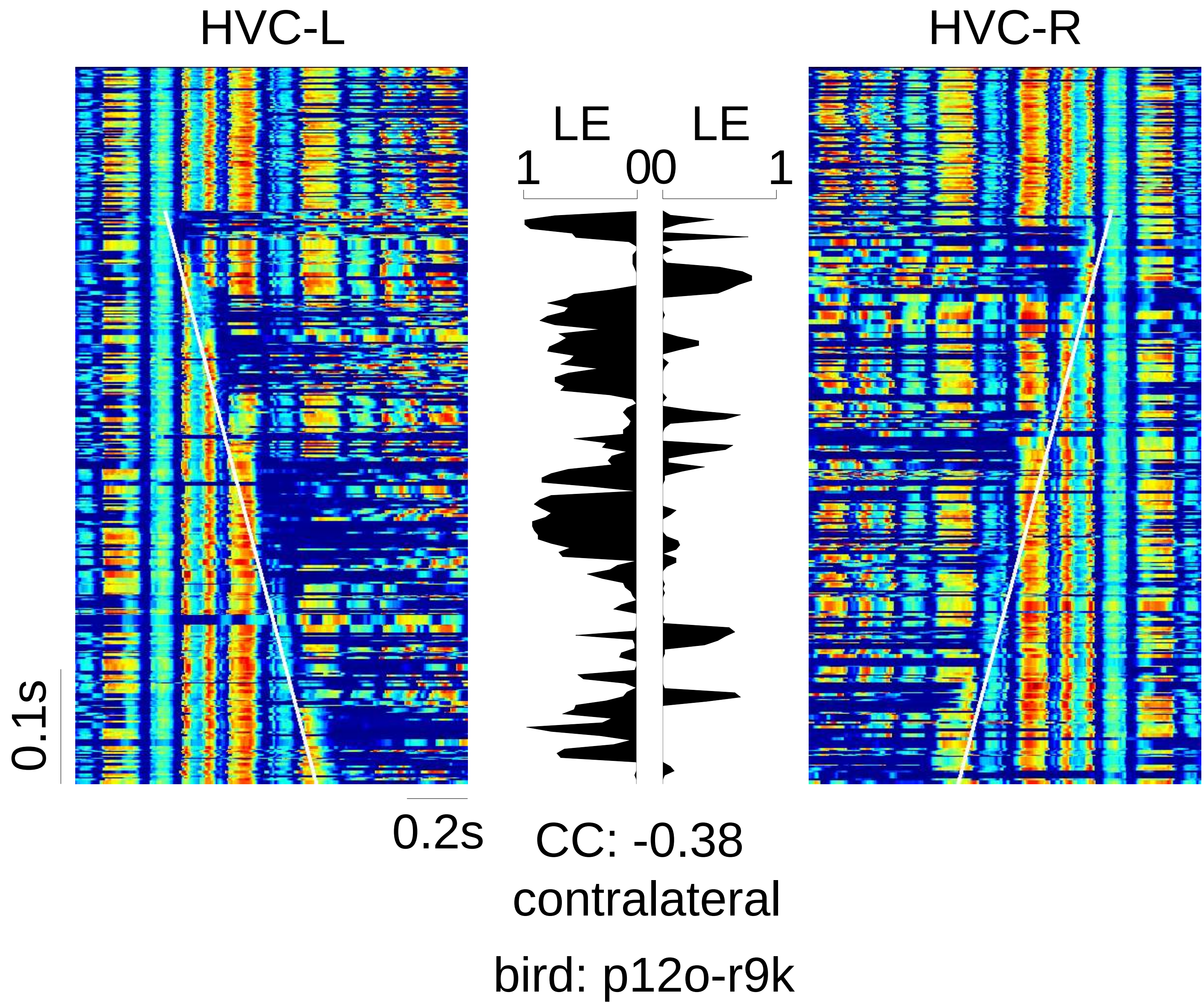
HVC-L
HVC-R
LE
LE
1
00
1
0.1s
0.2s
CC: -0.38
contralateral
bird: p12o-r9k

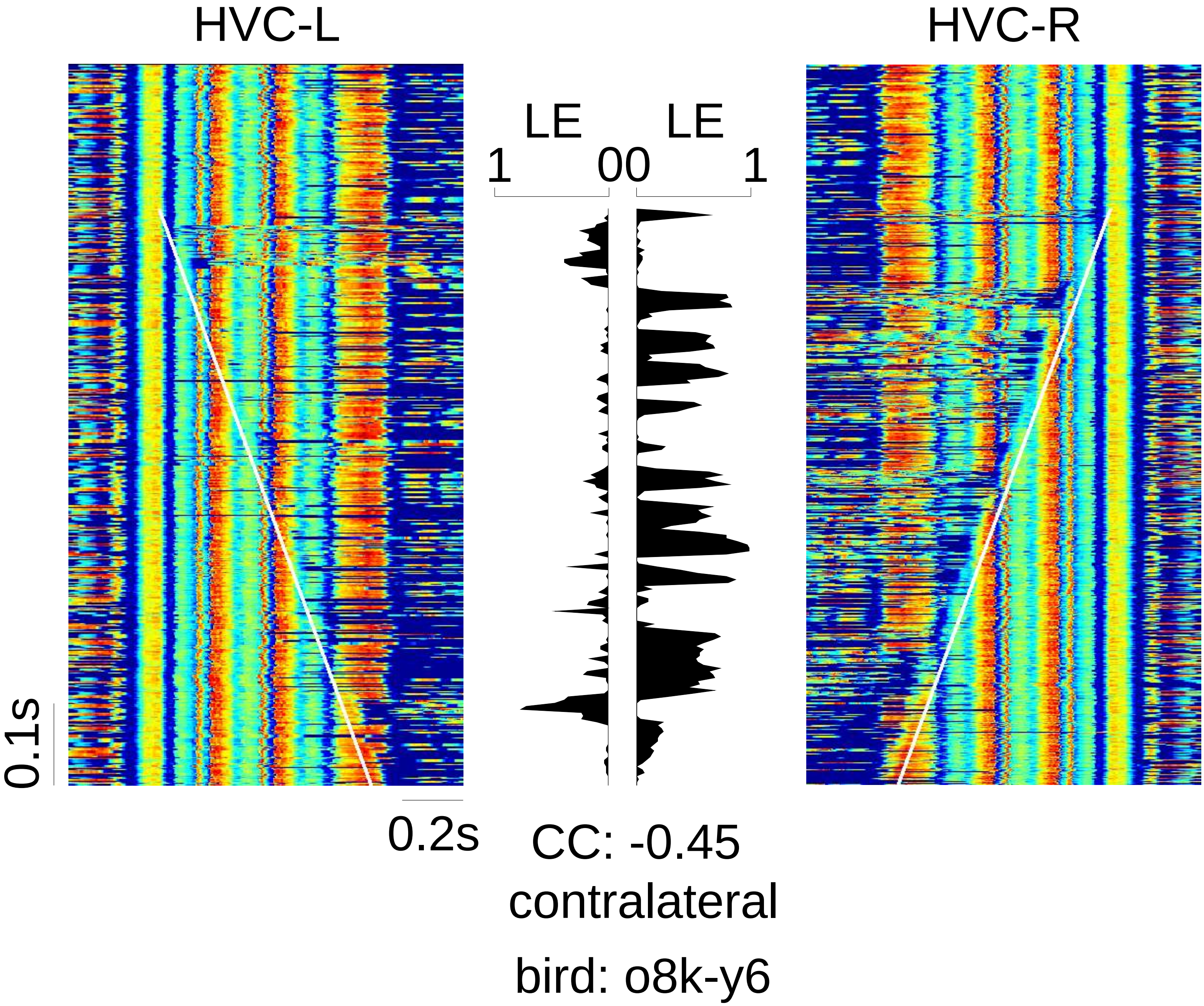

HVC-L
HVC-R
LE
LE
1
00
1
0.1s
0.2s
CC: -0.45
contralateral
bird: o8k-y6

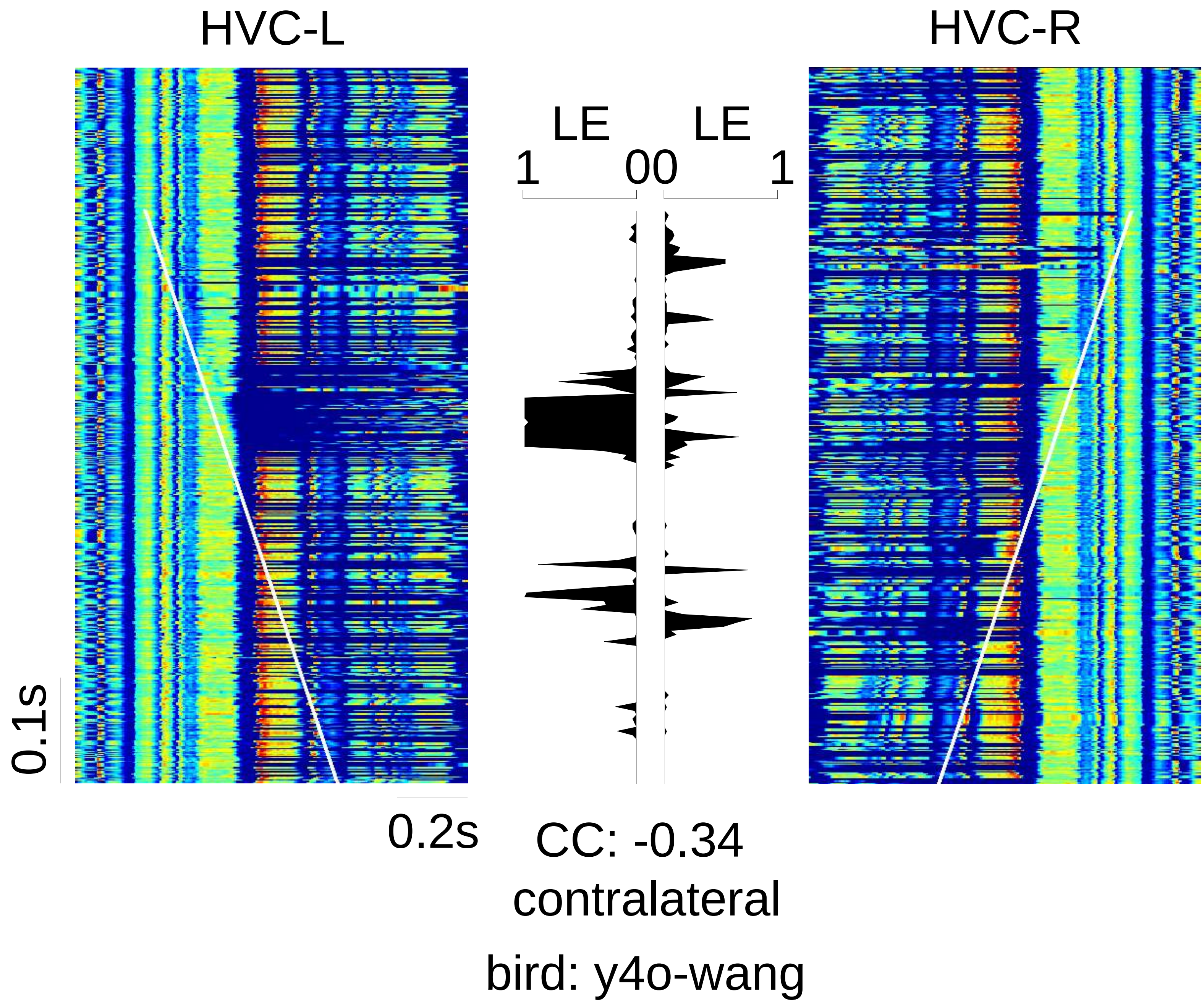

HVC-L
HVC-R
LE
LE
1
00
1
0.1s
0.2s
CC: -0.34
contralateral
bird: y4o-wang
