# Supplementary material for: Rapid Interhemispheric Switching during Vocal Production in a Songbird
Source: PLoS Biol. 2008 Oct 14;6(10):1–9. doi: 10.1371/journal.pbio.0060250 (PMC2567002; doi:10.1371/journal.pbio.0060250)
Supplement: Supplementary file 2 [file pbio.0060250.st001.doc]

**Table S1:** Percentages of HVC and LMAN stimulations that lead to syllable-level effects (truncations and distortions) and to motif-level effects (stoppings and restarts). ‘R’ and ‘L’ refer to the right and left hemisphere, respectively; ‘Short’ and ‘Long’ refer to the inter-electrode distance in HVC-implanted birds; and, the row labelled ‘StDev’ contains the standard deviations across birds.
