# Supplementary material for: Rapid Interhemispheric Switching during Vocal Production in a Songbird
Source: PLoS Biol. 2008 Oct 14;6(10):1–9. doi: 10.1371/journal.pbio.0060250 (PMC2567002; doi:10.1371/journal.pbio.0060250)
Supplement: Supplementary file 3 [file pbio.0060250.st002.doc]

**Table S2:** Complementarity of stimulation effectiveness in two hemispheres. Reported are the percentages of stimulation times that were associated with significant late effects (LEs) on sound amplitudes (first two columns). Also reported are the percentages of stimulation times associated with overlapping/non-overlapping LEs at the two stimulation sites (third and second-last columns), and the cross-correlations (CCs) of the associated LE curves (last column). Percentages for each bird are indicated relative to the set of all stimulation times. Labels are as defined in the legend of Table S1.
